# An ANN-based advancing double-front method for automatic isotropic triangle generation

**DOI:** 10.1038/s41598-022-16946-1

**Published:** 2022-07-30

**Authors:** Peng Lu, Nianhua Wang, Xinghua Chang, Laiping Zhang, Yadong Wu, Hongying Zhang

**Affiliations:** 1 Stake Key Laboratory of Aerodynamics, China Aerodynamics Research and Development Center, Mianyang, 621000 China; 2 School of Information Engineering, Southwest University of Science and Technology, Mianyang, 621010 China; 3 School of Intelligent Manufacturing Engineering, Chongqing University of Arts and Science, Yongchuan, 402160 China; 4 Unmanned Systems Research Center, National Innovation Institute of Defense Technology, Beijing, 100071 China; 5 School of Computer Science and Technology, Sichuan University of Science & Engineering, Yibin, 644005 China

**Keywords:** Aerospace engineering, Applied mathematics

## Abstract

The advancing front method (AFM) is one of the widely used unstructured grid generation techniques. However, the efficiency is relatively low because only one cell is generated in the advancing procedure. In this work, a novel automatic isotropic triangle generation technique is developed by introducing an artificial neural network (ANN) based advancing double-front method (ADFM) to improve the mesh generation efficiency. First, a variety of different patterns are extracted from the AFM mesh generation method and extended to the ADFM method. The mesh generation process in each pattern is discussed in detail. Second, an initial isotropic triangular mesh is generated by the traditional mesh generation method, and then an approach for automatic extraction of the training dataset is proposed. The preprocessed dataset is input into the ANN to train the network, then some typical patterns are obtained through learning. Third, after inputting the initial discrete boundary as initial fronts, the grid is generated from the shortest front and adjacent front. The coordinates of the points contained in the dual fronts and the adjacent points are sent into the neural network as the grid generation environment to obtain the most possible mesh generation pattern, the corresponding methods are used to update the advancing front until the whole computational domain is covered by initial grids, and finally, some smoothing techniques are carried out to improve the quality initial grids. Several typical cases are tested to validate the effectiveness. The experimental results show that the ANN can accurately identify mesh generation patterns, and the mesh generation efficiency is 50% higher than that of the traditional single-front AFM.

## Introduction

Mesh generation is one of the most important issues in numerical simulations, especially for complex engineering applications, because mesh generation is the first step for obtaining the numerical solution, and the mesh quality directly affects the accuracy of numerical solutions. So it plays an important role in numerical simulations, and it is still a grand challenge in engineering applications, despite several decades of effort^1–3^.

Generally, there are two main types of computational mesh, structured and unstructured grid. Compared with structured grids, unstructured grids have the following significant advantages: (1) Unstructured grids have the advantages of generality and flexibility to deal with complex geometries, and it is easy to control the size of the mesh cells and the density of mesh points. (2) The random data structure of the unstructured grids is conducive to mesh adaptation, therefore, unstructured grid generation requires little user time and effort. Additionally, the unstructured grid generation methods are well suited to inexperienced users because they require little user input.

Due to the outstanding advantages of unstructured grids, many scholars have proposed various unstructured grid generation methods. The widely used methods include the advancing front methods (AFM)^4–7^, Delaunay-based methods^8–11^, and the modified quadtree/octree methods^12–14^.

The advancing front methods cut the boundary of the computing domain into many discrete fronts and all the fronts form an initial front stack. Generally, the fronts are a set of edges in two dimensions (2D) or triangular faces in three dimensions (3D). During the advancing procedure, the original fronts become gradually the interior faces of the generated cells and a new set of fronts is created, this process continues until the computational domain is filled. Since the advancing procedure is carried out from the chosen front one by one, so the efficiency is relatively low for large-scale grid generation.

The majority of Delaunay-based methods mainly utilize the incremental algorithm, which starts with an initial triangulation of several points covering the whole computational domain. After inserting new points according to a step size limit, local triangulation is reconstructed based on the well-known Delaunay criterion until the whole triangular grids are generated to meet the grid size distribution requirement. The efficiency is relatively high since several cells are generated simultaneously. However, special treatment should be carried out carefully to confirm the original geometry.

The idea of the modified quadtree/octree algorithms can be divided into the following steps. Firstly, the whole computational domain is covered with a multi-level rectangular (2D)/cubic (3D) tree structure. Secondly, according to the shape and flow field characteristics, the local mesh is subdivided by the tree structure until the grid size meets the computing requirements. Finally, the cells outside the boundaries are deleted, and the cells that intersect the boundary are cut into polygons or polyhedrons, then the polygons or polyhedrons can be further cut into simplex, e.g. triangles or tetrahedrons.

After several decades of effort by CFD researchers, unstructured grid generation techniques have made great progress. However, as we know, all the unstructured grid generation methods have their advantages and disadvantages. As mentioned in NASA’s CFD vision 2030 study^15^, a high-quality automated mesh generation over complex configurations is still a grand challenge in CFD engineering applications. Even with modern commercial software (e.g., Ansys ICEM-CFD, Fluent Meshing, Hypermesh, and Pointwise) and open-source packages (e.g., NetGen and Gmsh), automation level and mesh quality are two of the most important issues in mesh generation. According to reference^16^, mesh generation usually takes up about 60% of the manpower time in the entire computation cycle, a highly automated mesh generation method can undoubtedly save a lot of labor costs in CFD applications.

To improve the traditional unstructured grid generation algorithms, some researchers tried to introduce artificial intelligence (AI) algorithms such as expert systems and neural networks to enhance the automation level of mesh generation and had obtained some preliminary results. For example, Dolšak and Jezernik proposed an expert system to decide the appropriate resolution for mesh generation, this expert system was used to create rules of mesh generation to replace previous expertise^17^. Yao et al. used an ANN-based element extraction method to generate a mesh automatically^18^, the ANN was used to represent some element extraction rules and to train the relationship behind these rules. Yilmaz and Kuzuoglu proposed a particle swarm optimization method to optimize the quality of the hexahedral mesh, which solved the problem of the poor effect of the Laplacian mesh smoothing algorithm in concave regions^19^. In the past year, machine learning algorithms based on neural networks have been further developed in grid generation and optimization, which has further improved the level of grid intelligence^20–23^. Pan et al.^24^ solved the self-learning problem of grid generation by combining reinforcement learning with supervised learning. Papagiannopoulos et al.^25^ proposed a triangular mesh generation method with ANNs. They trained three ANNs to predict the number of candidates to form a triangular element, the coordinates of those vertices, and their connection relations with existing segments on the boundary, respectively. A limitation of their method is that the overall mesh quality is limited by the training data, and it cannot be applied to arbitrary and complex domain boundaries because of the fixing number of boundary vertices in the model input. In recent years, end-to-end mesh generation methods based on the neural network are gradually used to solve the problems of rendering in graphics^26,27^. However, these methods lack the corresponding flexibility for computer-aided engineering (CAE) grid generation, such as local refinement in some areas and coarsen mesh in others. These earlier works should be continuously improved forward, especially with the rapid development of AI technology. To the authors’ knowledge, there is still no mature application of AI algorithms in high-quality automatic mesh generation. In our previous work^28^, the authors have tried to develop an ANN-based AFM to improve the computational efficiency of unstructured grid generation. However, although some AI technologies have been introduced into unstructured grid generation and the efficiency is improved to a certain degree, only one front is advanced in each advancing step, so the efficiency of mesh generation can be further improved if two or more fronts are advanced simultaneously in each advancing step. That is the main point of this work. Therefore, in this paper, combining the advantages of ANN and AFM, an ANN-based advancing double-front method (ADFM) is proposed. In this way, a large number of geometric operations (tedious intersection checking with the existed fronts, the selection of active fronts, and candidate points) will be reduced to improve mesh generation efficiency.

The rest of this paper is organized as follows. Section ‘Related work and advancing double-front method’ briefly reviews the traditional AFM and ANN and introduces the idea of ADFM. In section ‘ANN-based advancing double-front method’, the new isotropic triangle mesh generation technology (ANN-based ADFM) will be introduced in detail, including the extraction of the training data set, preprocessing of the training data, training of ANN, mesh size controlling, and quality optimization of initial mesh. In section ‘Results and discussion’, several typical cases, such as the NACA0012 airfoil and the 30P30N three-element airfoil, are tested to validate the effectiveness. Finally, some concluding remarks are summarized and future works are discussed in section ‘Conclusions’.

## Related work and advancing double-front method

### Brief introduction of traditional advancing front method and its problem

Adopting AFM to generate triangular grids was first formulated by George^4^, but this original publication did not receive significant immediate attention. The first journal publication of this method was proposed by Lo^5^. Subsequently, many researchers extended this method to 3D mesh generation^6,7^.

**Figure 1:**
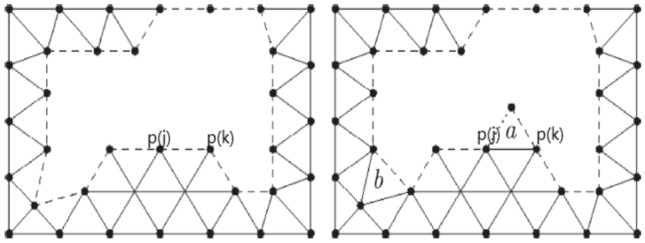
Schematic diagram of mesh generation by AFM.

The discrete fronts are some line segments (called edges) in 2D cases that distinguish the meshed area from the unmeshed area as shown in Fig. 1. At the beginning of the mesh generation process, the initial fronts consist of the sequence of edges that connect consecutive boundary points. When new triangles are generated one by one, the meshed area continues to advance, and the unmeshed area shrinks simultaneously. When the data stack of the front is empty, it indicates that the whole computing domain has meshed, and the mesh generation procedure is completed. As shown in Fig. 1, all the dashed lines represent the fronts, the segment **[*P*(* j* )*, P*(* k* )]** is chosen as an active front to generate a new triangle.

**Figure 2:**
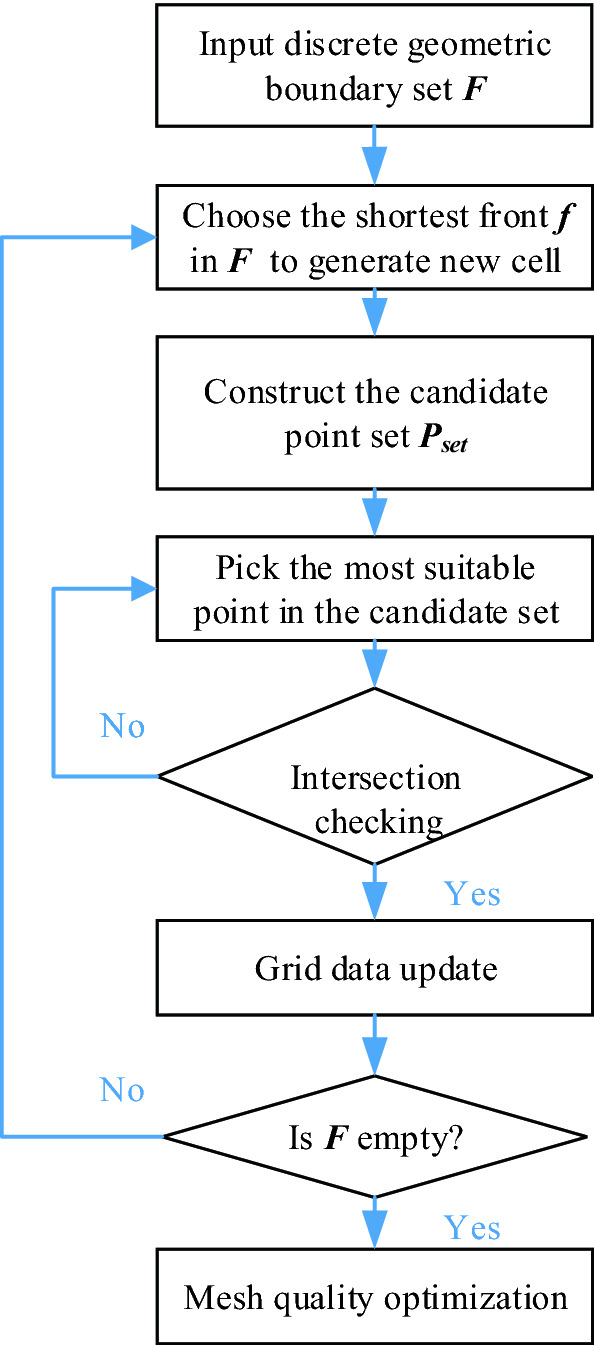
Flowchart for triangular mesh generation by the traditional AFM.

As shown in Fig. 2, the procedure of the traditional AFM can be summarized in the following steps:

1. Input the original discrete geometric boundary, such as the discrete rectangle in Fig. 1. All the discrete edges are called the set of initial fronts, ***F***.
2. When ***F*** is empty, skip to Step 6; otherwise, search for the most suitable front ***f ∈ F*** as the active front to generate a new cell, usually the shortest one is chosen as the candidate.
3. Search the points near the selected front, ***f***, to obtain a set of candidate points to generate a triangle. The candidate point set ***P***_***set***_ contains the newly created point ***P***_***best***_ and the endpoints of some adjacent fronts. The position of ***P***_***best***_ = ***P***_***m***_ + ***S***_***p***_⋅***n***_***ab***_, where ***P***_***m***_ represents the center point of the front ***f***, ***S***_***p***_ represents the advance distance at ***P***_***m***_, and ***n***_***ab***_ is the unit normal vector pointing to the computational domain. After the candidate point set ***P***_***set***_ is created, and then sort these points by the mesh quality coefficient of the cells to be generated. To choose the points that already existed on the adjacent fronts, the quality coefficient of ***P***_***best***_ needs to be discounted.
4. Pick the best candidate point ***P***, ***P ∈ P***_***set***_, and connect with the active front ***f*** to form a new possible cell, ***Cell***_***t***_.
5. If ***Cell***_***t***_ passes through the validity checking such as intersection judgment, then add this cell into the generated mesh list, update the active front ***F***, then go back to Step 2; otherwise, go back to Step 4 for the next candidate point.
6. Mesh quality optimization to improve the quality of the final mesh.

It is obvious from Fig. 2 that the necessary step is intersection checking in AFM, there will be a large number of searching and sorting operations for each new cell generation, so the advancing efficiency needs to be further improved.

### Formulation of the improved advancing double-front method

**Figure 3:**
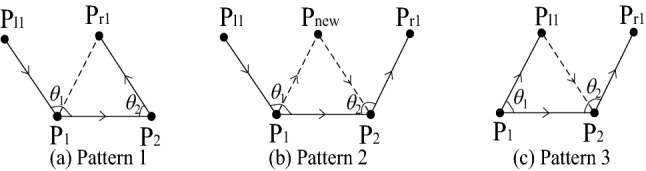
Three basic patterns of the input–output relationship.

To reduce the searching and sorting operations in traditional AFMs, the fronts around the active front are taken into account as the environment of mesh generation^29^. According to the angles *θ*_1_ and *θ*_2_ between the fronts, it can be concluded that there are three patterns of isotropic triangles generated by the traditional AFM as shown in Fig. 3.

In Fig. 3b, where the solid line connected by ***P***_**1**_***P***_**2**_ is the active front which will generate a new triangle (***P***_**1**_***P***_***new***_***P***_**2**_), ***P***_***l1***_ and ***P***_***r1***_ are the directly adjacent points on the left and right ends of the active front ***P***_***1***_***P***_***2***_. It should be noticed that the solid lines ***P***_***l1***_***P***_***1***_ and ***P***_***2***_***P***_***r1***_ belong to the inactive fronts which constitute the external environment for the active front ***P***_***1***_***P***_***2***_. Once a triangle is generated, the new active front should be stored by the right-hand rule, to ensure that the mesh generated by this new front grows towards the computational domain. It is obvious that pattern **1** and pattern **3** directly connect existing points without generating a new point. Therefore, in this way, some intersection checks can be reduced to improve efficiency. The following lists the rules in the system to distinguish between different patterns.

1 $$\begin{gathered} \theta_{1} \ge 90^{^\circ } ,\,\,0 < \theta_{2} \le 90^{^\circ } \,\,\,\,\, \mathrel\backepsilon \,{\text{pattern }}1 \hfill \\ \theta_{1} \ge 90^{^\circ } ,\,\,\theta_{2} \ge 90^{^\circ } \,\,\,\,\,\,\,\,\,\,\,\, \mathrel\backepsilon \,\,{\text{pattern }}2 \hfill \\ 0 < \theta_{1} < 90^{^\circ } ,\,\,\theta_{2} > 90^{^\circ } \,\,\,\, \mathrel\backepsilon \,\,{\text{pattern }}3 \hfill \\ \end{gathered}$$

**Figure 4:**
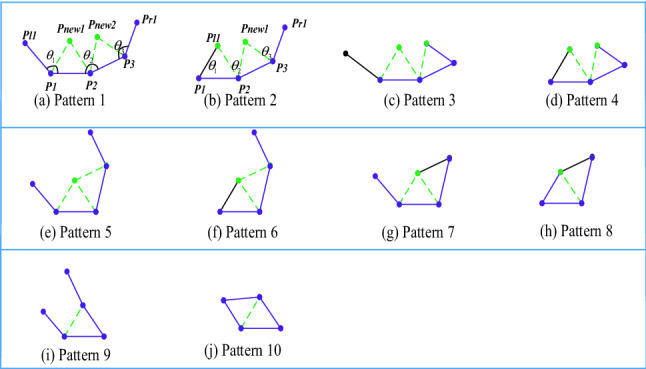
Ten patterns of the input–output relationship for advancing double-front method.

Inspired by the above ideas, to further improve the efficiency of mesh generation, a novel method based on double-front advancing simultaneously is proposed in this paper. As shown in Fig. 4a, the edge ***P***_**1**_***P***_**2**_ is the first active front and ***P***_**2**_***P***_**3**_ is the second one, which means that each of them will generate a new triangle. ***P***_***l1***_ and ***P***_***r1***_ are the directly adjacent points on the left and right ends of the two fronts. However, this is only a special case of the ADFM to generate two new grids. To improve grid generation automation, the environment for grid generation needs to be classified. According to the value of *θ*_2_, grid generation can be divided into three categories, corresponding to the three rows in Fig. 4, respectively. When *θ*_2_ > *θ*_max_, there will generate two triangles that do not share one edge, such as patterns 1 ~ 4 in the first row; in other cases, when *θ*_min_ ≤ *θ*_2_ ≤ *θ*_max_, two new triangles will also be generated, but they will share one edge, such as patterns 5 ~ 8 in the second row; finally, when *θ*_2_ < *θ*_min_, the two active fronts will generate only one triangle. Here *θ*_max_ and *θ*_*min*_ are constants, in this paper, *θ*_max_ is 170 degrees and *θ*_min_ is 70 degrees. The above only discussed the influence of different sizes of *θ*_2_ on the procedure of grid generation, and the angles *θ*_1_ and *θ*_3_ between the active front and its neighbors also determine the details of mesh generation. For example, in pattern 4 in the first row, where *θ*_1_ < *θ*_min_ and *θ*_3_ < *θ*_min_, in this case, two new triangles will be generated, however, only two new edges will be generated and no point generated. The grid generation approach for other patterns is similar to the one described above. Therefore, all the external environments of mesh generation can be divided into 10 patterns according to the angles (*θ*_1_, *θ*_2_, *θ*_3_). It should be noted that the case where *θ*_2_ is greater than 180° is not shown in the figure, which is the same as patterns 1–4 and two triangular elements will be generated.

Similar to the mesh generation patterns of single front in Fig. 3, the classification of double-front advancing at the same time will be more complex. So to establish such a set of rules would be cumbersome, and sometimes, a fixed rule will lead to a poor quality mesh. Meanwhile, these rules are usually obtained through an interactive trial-and-error process which depends on the experience. An automatic method of obtaining rules is essential and even indispensable for this method to be applied to a wider range of problems. Due to the ability to approximate any complex nonlinear relationship and self-learning, the well-known ANN-based machine learning method is introduced here to solve the above problem.

**Figure 5:**
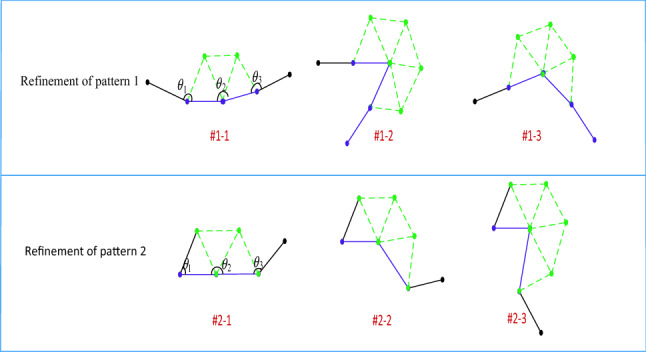
Refinement of some patterns based on knowledge rules.

In the procedure of grid generation, the probability of occurrence of the above 10 patterns must be different. For example, the probability of occurrence of pattern 1 is higher than that of pattern 10, these will be discussed in detail in Sect. Mesh quality and time‑consuming assessment. Because of the high probability of the occurrence of pattern 1, it is necessary to refine pattern 1 to further improve efficiency. Zhang et al. proposed a concept, the combination of symbolism and connectionism, for the third generation of artificial intelligence^30^. According to this method, in this work, the ANN is utilized to do the initial classification, and then the knowledge rules are adopted to subdivide some patterns. As shown in Fig. 5, the core idea is to generate the new triangular cells based on the ANN according to the size of *θ*_2_. This method can reduce the difficulty of neural network design and maximize the efficiency of grid generation.

### Brief introduction of artificial neural network (ANN)

**Figure 6:**
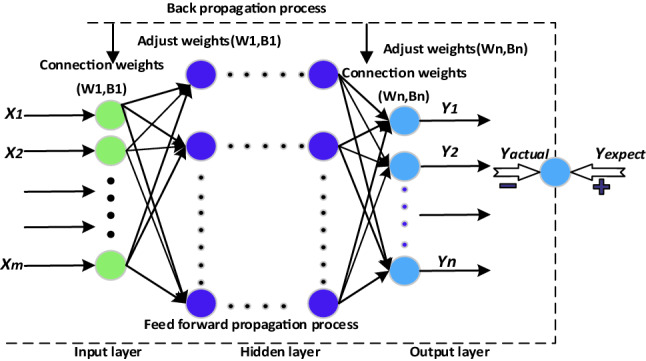
Schematic diagram of BP-ANN.

In the following context, the widely-used ANN model will be applied to ADFM, so here we give a brief introduction to ANN. As we know, ANN is a powerful tool in data processing, just like neurons in the brain, consisting of calculation units that imitate neurons and weighted connections between neurons which are called weights^31^. It can minimize the error between predicted output and ideal output through continuous data learning. As shown in Fig. 6, a typical ANN architecture usually contains three parts: an input layer, several hidden layers, and an output layer. The neural network receives input data in the input layer and predicts output data in the output layer. It can be regarded as a general approximation of an unknown complex function^32^.

It has been proven that if there are enough hidden units and the weight parameters are appropriate, the neural network can approach the nonlinear function with arbitrary accuracy^31^. By training the model, iterations are made to adjust the weights between units. The training process consists of two phases. First, the data is propagated forward through the network, and the error loss between the desired output and the output is calculated. Then, machine learning algorithms such as gradient descent are used to adjust the weights between each connection layer to reduce the error.

## ANN-based advancing double-front method

### Description of the algorithm

To improve the efficiency of the traditional single-front AFM method in generating the isotropic triangles, the idea of ADFM is proposed in section ‘Formulation of the improved advancing double-front method’. However, the environment around the fronts to be advanced is complex and changeable, and the classification process needs large human judgment. So the multi-classification method of ANN is adopted here to identify and classify the external environment in this study, which is helpful to improve the intelligence level of mesh generation.

**Figure 7:**
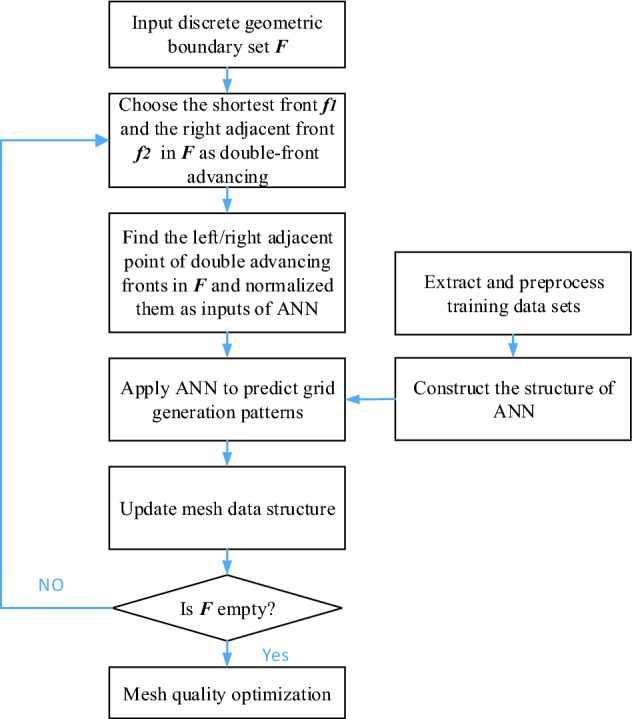
Flowchart for generation isotropic triangular mesh by ANN-DAFM.

The procedure of the ANN-based ADFM can be summarized in Fig. 7. An ANN model is trained to establish the mapping relationship between inputs and outputs. Training neural network model is essential to determine the weights matrix and the bias terms between neurons in different layers. This new method consists of the establishment of a neural network and mesh generation. The samples of ANN training are extracted from mesh data generated by traditional methods, the trained model is utilized to predict patterns in mesh generation.

### ANN model design

The design of a neural network directly affects the performance of the algorithm. The ability of ANN is greatly dependent on the architecture. The input of ANN is the coordinates of five points, the two fronts to be advanced and the points on the left and right adjacent fronts, the output layer is designed to solve the 10 classifications of mesh generation. For a multi-classification neural network system, the one-hot encoding (OHE) is used because all states are equidistant without bias in Euclidean space^33^. The logistic regression model can be expressed as follows:

2 $$h(x) = \frac{1}{{1 + e^{{ - (w_{1} x_{1} + w_{2} x_{2} + ... + w_{n} x_{n} )}} }}$$

where the value of the function is 0–1, x_1_, x_2_, …, x_n_ are the components encoded by OHE, and w_1_, w_2_, …, w_n_ are the weights of the corresponding components.

In the training stage of ANN, an error will be sent backward through the backpropagation process to optimize the weights and biases of the ANN, the error is defined by the mean square error (***MSE***) which defined as

3 $$MSE = \frac{1}{n}\sum\nolimits_{i = 1}^{n} {(y_{i} - k_{i} )^{2} }$$

where ***y***_***i***_ and ***k***_***i***_ are the network output value and the target expected value respectively, ***n*** is the total number. When the ***MSE*** reaches a minimum or training steps reached the set value, the training process is finished.

For the hidden layer design, although there are some works contributed in this area^34^, there is still no mature way to determine the number of hidden layers in an ANN and the number of neurons in each hidden layer for optimal architecture. In this work, the architecture of ANN is designed by the traditional empirical method with 10 neurons on the input layer, 10 neurons on the output layer, two hidden layers with 25 neurons in the first hidden layer, and 18 neurons in the second hidden layer.

### Extraction and preprocessing of training sample data

**Figure 8:**
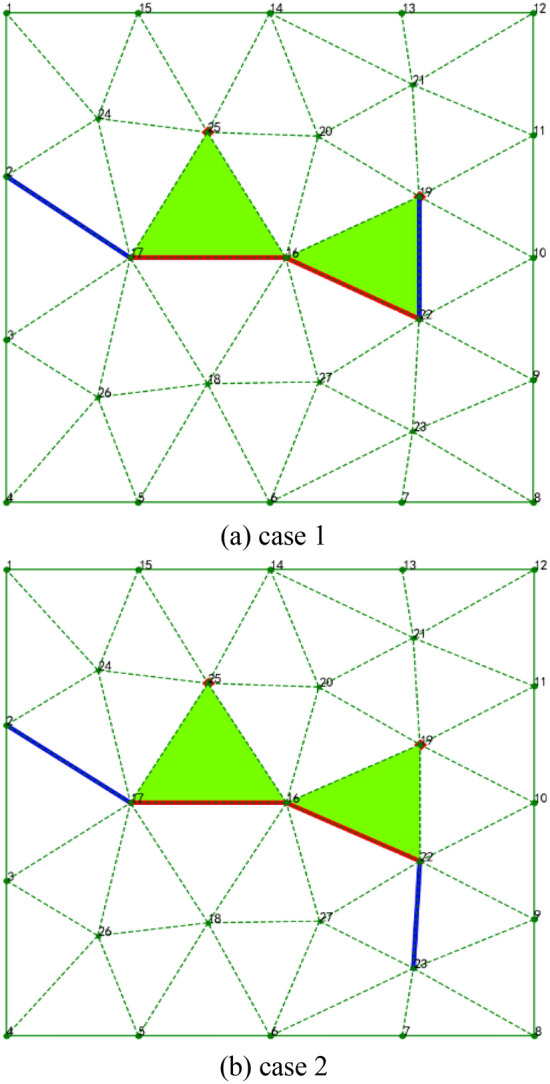
Extract training samples generated by the traditional method.

The purpose of ANN is to learn the rules of mesh generation from existing mesh data generated by some traditional methods such as the Delaunay method and AFM, and so on. In this work, a variety of complex geometry mesh data are used to extract training data. For the convenience of introduction, a simple shape is used to describe the process of training data extraction. As shown in Fig. 8, there are two training samples. The red lines represent the fronts to be advanced, according to the right-hand rule, the first active front is composed of Point 17 and Point 16, denoted as Front^16,17^, the second active front denoted as Front^16,22^, the red points (Point 25 and Point 19) are mesh points advanced by the corresponding active fronts. The blue lines represent the left and right adjacent fronts of the double fronts. In Fig. 8a, case 1 corresponds to pattern 3 in Fig. 4, in this case, the first active front will generate a new mesh point (Point 25) and the second active front will use the existing point to form a triangle (Triangle^16,19,22^) with no new point generated. In Fig. 8b, case 2 is different from case 1 in which it generates two new points, this case belongs to pattern 1 in Fig. 4.

**Figure 9:**
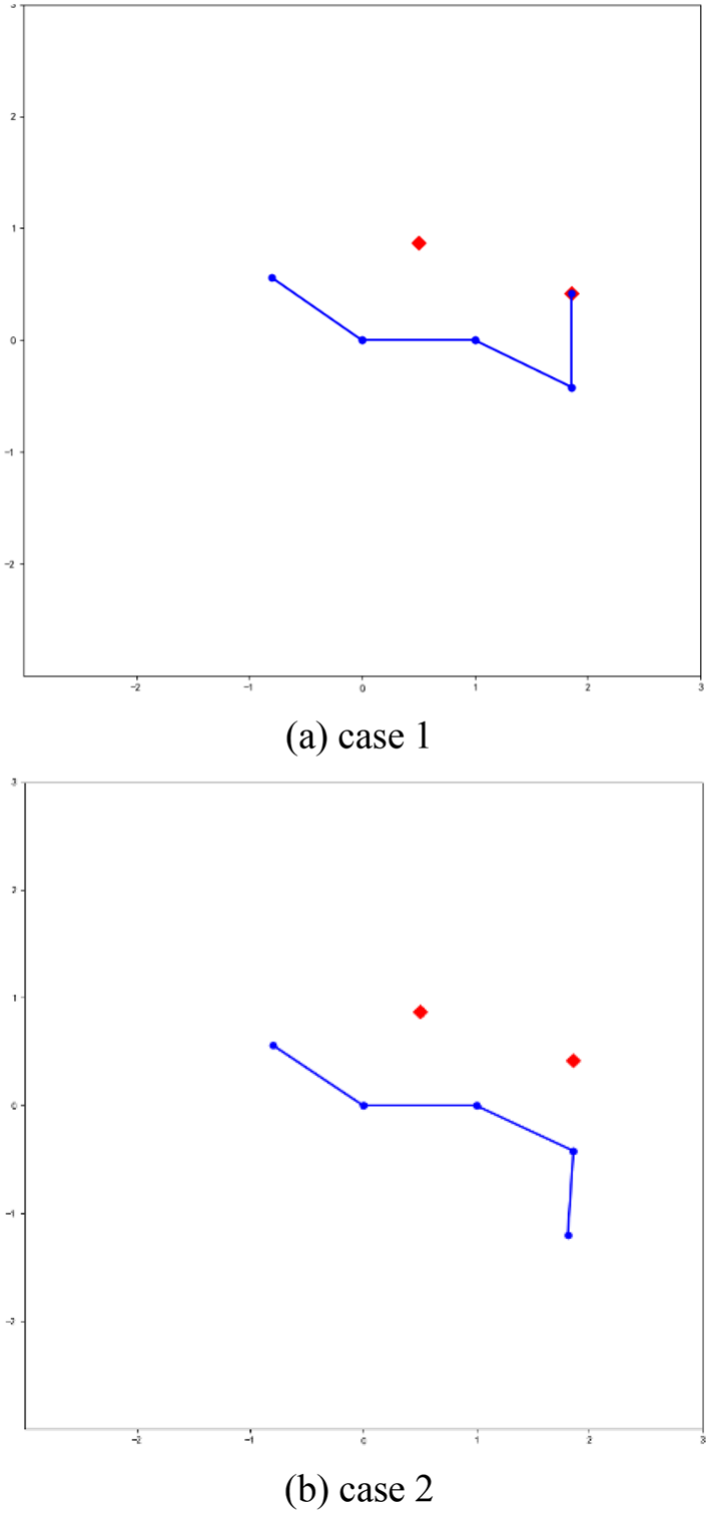
Normalized data of the two extracted training samples.

As shown in Fig. 8, the input data of ANN are the coordinates of five points on the active fronts, and the differences in these coordinate values are very large, some of them can reach several orders of magnitude, which are not beneficial to the convergence of ANN. Therefore, it is necessary to preprocess the training data. The most popular method of data preprocessing is data normalization, which is used to reduce data with different representations to the same scale. The common scale ranges are [−1, 1] and/or [0, 1]. For ANN or support vector machine (SVM), it is essential to use normalization in data preprocessing. Of course, data normalization is not required for some machine learning methods, such as decision trees. In this study, the transition, scaling, and rotation operations are used for data normalization^35^, and Fig. 9 presents the normalized data graphs of the two extracted training samples in Fig. 8.

### The training, verification and test of ANN

The experiment was implemented on a computer with an Intel Core i5-3230 M, 16 GB of random memory, and a graphics card with 4 GB. An open-source machine Learning Framework, TensorFlow, was used to train the network parameters of the proposed mesh generation pattern recognition algorithm. The software environment is as follows: WIN10, Tensorflow-1.10, and Python-3.6 from the Anaconda-3 distribution to create an independent Python environment.

**Figure 10:**
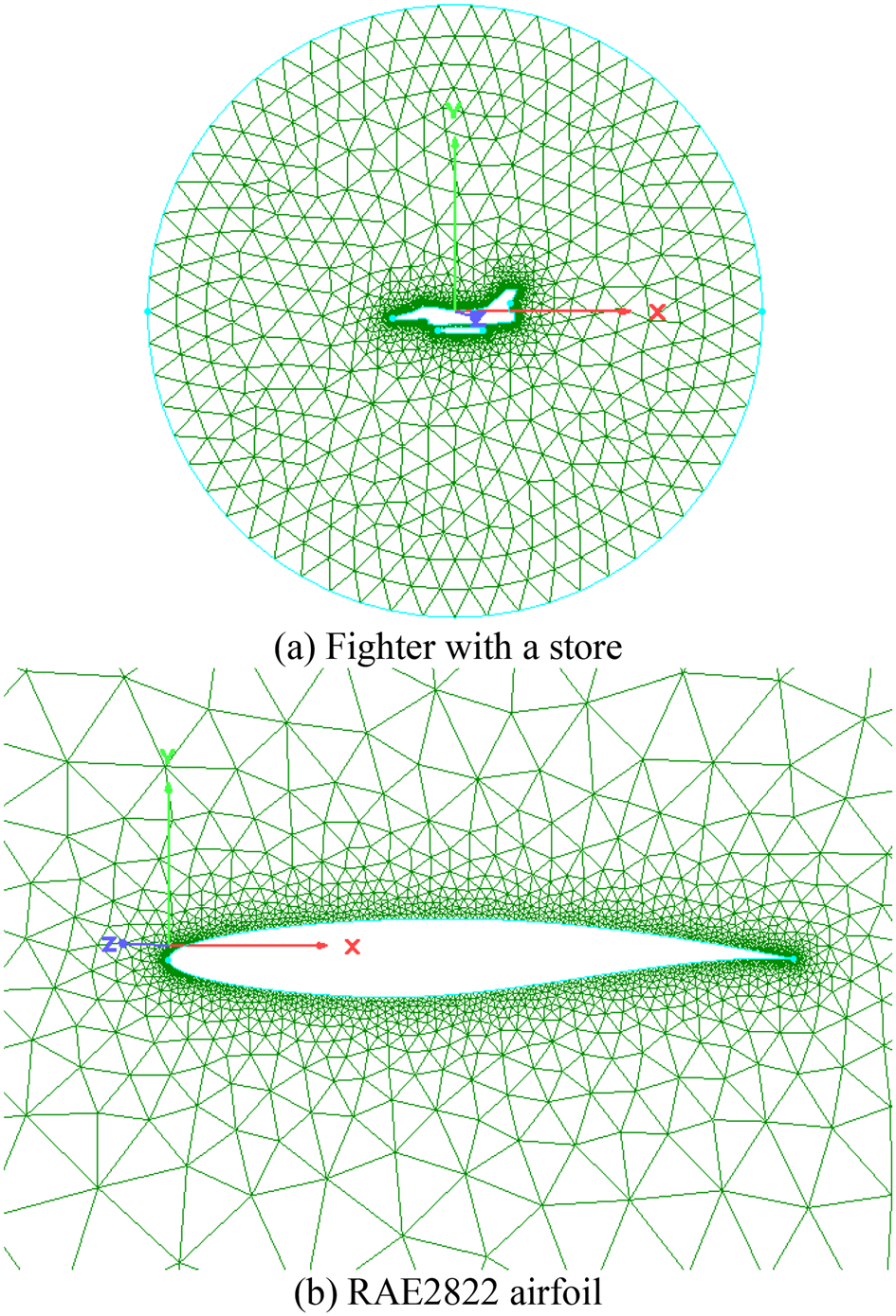
Training samples generated by the traditional AFM.

Figure 10 shows a 2D mesh over a fighter with a store and a RAE2822 airfoil as training sample data, the numbers of the isotropic triangles are 8708 and 10,352 respectively, any two connected edges and their left and right adjacent points will be used as a set of training data. The mesh training samples were randomly divided into the training set, test set, and verification set according to the proportions of 70%, 15%, and 15%.

**Figure 11:**
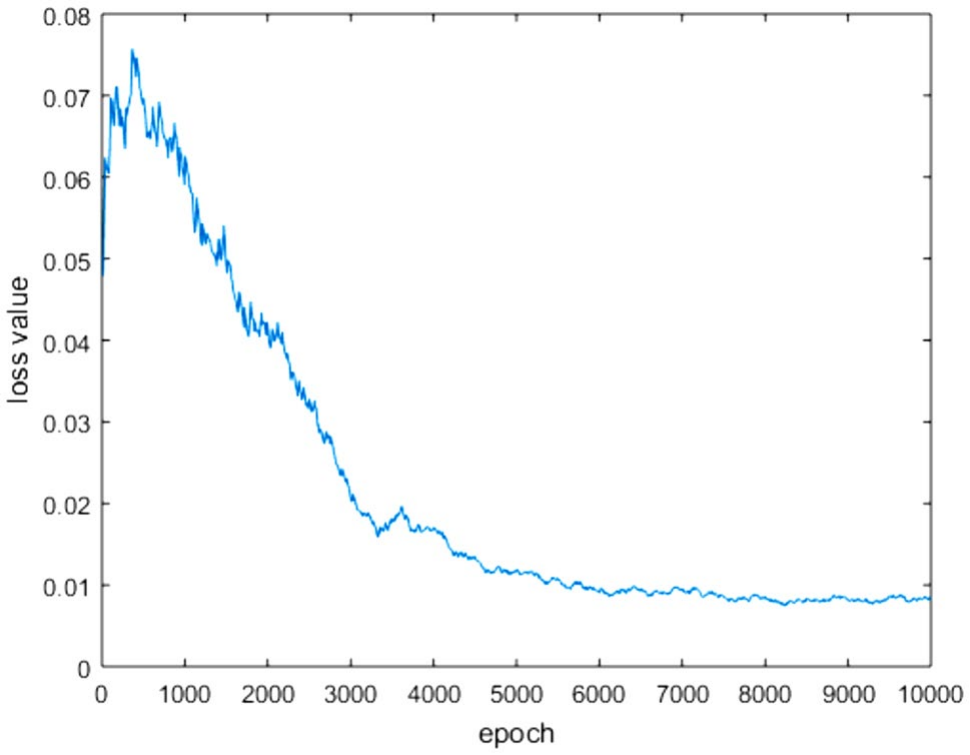
Loss of training dataset for each epoch.

The early stage of ANN design mainly consists of training and verification, the Adam optimization algorithm is used to minimize the loss function value during training. when the loss of the network is less than 1.0e^-4^, it indicates that the ANN has converged, and the weights and biases of all the layers will be saved as a model. At last, the test set will be utilized to check the prediction performance of the system. The verification is similar to the test process except that the test process does not adjust the weights and biases of ANN. Figure 11 shows that the loss value changes significantly and has a decreasing trend, indicating that ANN gradually converges and tends to be stable.

### Mesh size control

Mesh size control has a great influence on mesh quality and flow solution accuracy. It is usually expected that the grids should be refined in key areas such as geometric curvature and large flow gradient, while in the outer far-field, grids can be very large since the flow gradient is almost zero, and the transition from the solid wall to far-field should be as smooth as possible.

**Figure 12:**
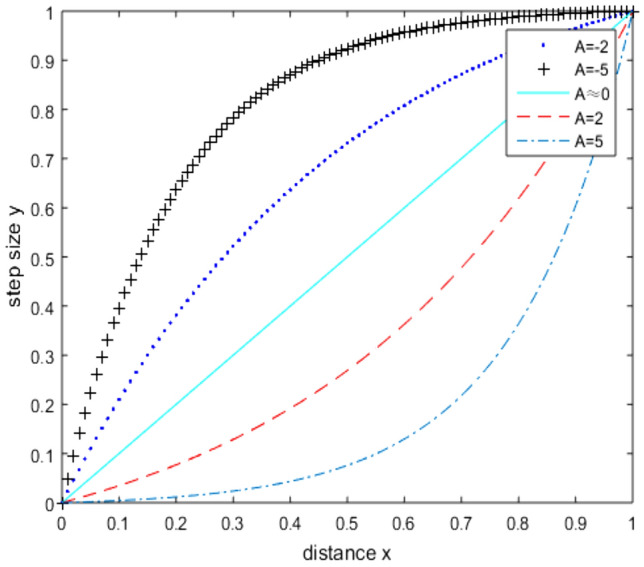
Relation between distance and mesh size.

Generally, the control methods of unstructured mesh size mainly include the background mesh method, interpolation method, function designation method, and adaptive method of flow field characteristics. In this study, the exponential function method is used to determine the mesh step size in the computational domain^36^. The mesh size can be written as:

4 $$y = \frac{{e^{Ax} - 1}}{{e^{A} - 1}}\,\,\,\,\,$$

where ***x*** is the distance between the active front and the nearest initial front on the wall, the parameter ***x*** has been normalized to between 0 and 1, and ***y*** (0 ≤ ***y*** ≤ 1) is the normalized mesh size calculated for the corresponding front, the desired mesh size is obtained by the anti-normalization process of ***y***. In the actual mesh generation process, the mesh size of the first layer and the far-field boundary should be given, the Newton–Raphson iteration method is used to obtain the value of ***A***. Figure 12 shows the relationship between the distance ***x*** and mesh size ***y*** with different values of ***A***. It should be noted that when x = 0, the mesh size needs to be given separately according to the current fluid environment and the geometry considered.

### Mesh quality enhancement options

**Figure 13:**
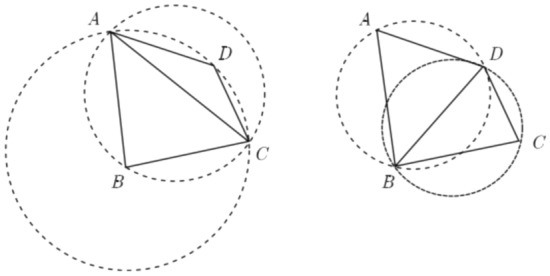
Diagonal transformation based on Delaunay criteria.

**Figure 14:**
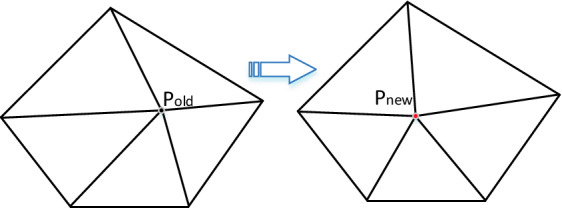
Mesh quality optimization by spring method.

Generally, the initial grids generated by ANN-based ADFM need to be optimized to improve mesh quality. In this paper, the Delaunay criterion is introduced to carry out diagonal transformation for elements with poor quality^37^, as shown in Fig. 13. Meanwhile, the spring relaxation method is introduced to optimize the coordinates of non-boundary nodes^38,39^, which improves the element’s regular shape by repositioning a node at the barycenter of the polygon formed by its neighbor elements, as shown in Fig. 14. Numerical experiments show that better mesh quality can be obtained by alternatively using the two optimization methods after several iterations.

## Results and discussion

Once the ANN has been trained, it can be used to automatically predict mesh generation patterns for other similar problems. To demonstrate the capability of the present ANN-based ADFM, some typical cases will be illustrated in this section.

### Special characters “AI + CFD”

**Figure 15:**
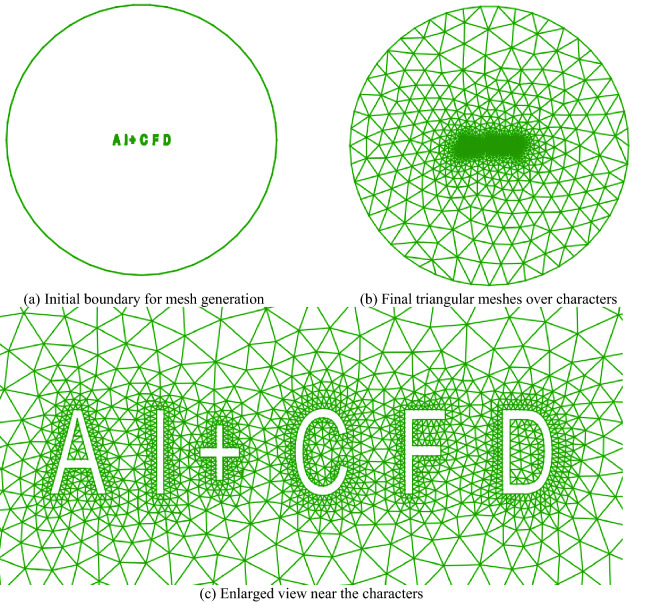
Isotropic triangular mesh over some special characters.

Figure 15 shows the isotropic triangular mesh over some special characters (“AI + CFD”), which contains many types of concave and convex angles. These characters are automatically generated by general computer-aided design software (CAD2021) and then discretized by Pointwise software. In this case, because the influence of the flow field is not considered, uniform distribution is adopted to discrete the object surfaces, the far-field size is 5 times that of the object surface. On the other hand, these characters are of multi-connected domains, and each character contains at least one closed connected domain. The ability of the present method to deal with multi-connected domains is verified. This mesh includes 1978 points and 3506 isotropic triangular cells in the computational domain. It can be seen that the mesh transition from the object surface to the far-field is reasonable, and a smooth mesh distribution around the characters can be obtained.

### NACA0012 airfoil

**Figure 16:**
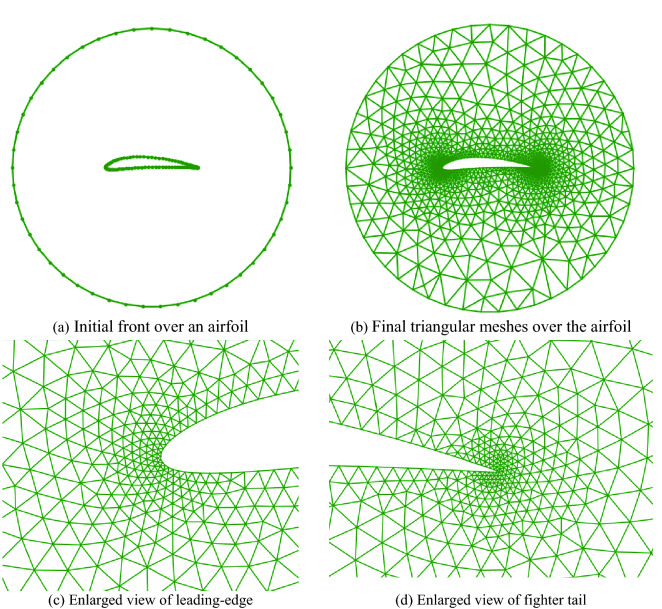
Mesh generation over an airfoil4.3 30P30N three-element airfoil.

Here, a more practical geometric shape (NACA0012 airfoil) is taken as an example. The computational mesh is shown in Fig. 16, which includes 1191 mesh points, 2234 triangular cells, and 3425 faces. There are 148 points at the solid wall and 49 points at the far-field boundary. The far-field boundary is designed as a circle and the center of the circle is the middle point of the airfoil. Thus, when the far-field boundary is rotated around its center, the solution field can remain unchanged. Because the gradient of the flow at the leading-edge and trailing-edge regions of the airfoil varies dramatically, more dense grids are needed to obtain the appropriate numerical solution, the mesh density in the leading and trailing edges is ten times that of the middle of the airfoil, a gradual transition is adopted from dense mesh area to sparse mesh area. In the whole computational domain, the mesh is smoothly transferred from the solid wall to the far-field circle.

### 30P30N three-element airfoil

**Figure 17:**
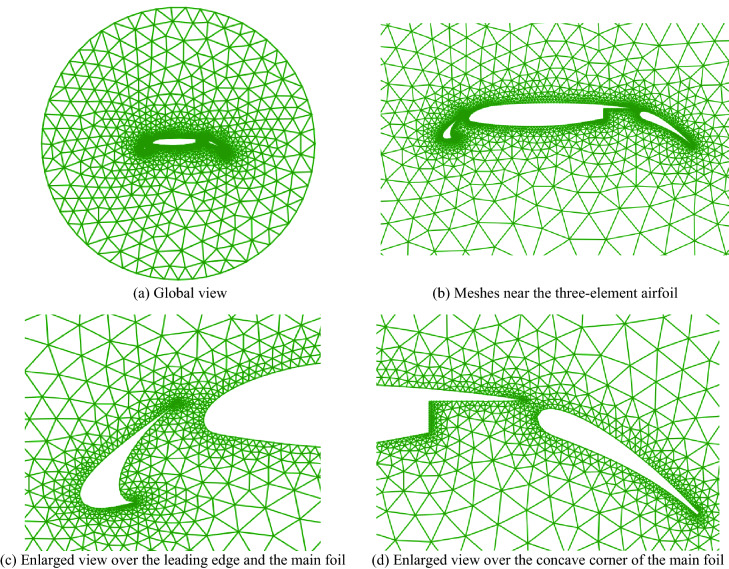
Triangular mesh generation over a three-element airfoil.

The last case is the well-known 30P30N three-element airfoil, which is designed by McDonnell-Douglas as a test case for aerodynamic prediction of three-element high-lift devices including slats and flaps. The final isotropic triangular grids are shown in Fig. 17, including 4206 triangular cells, 6534 faces, and 2326 points. There are 390 points at the geometry boundary and 59 points at the far-field boundary. It is similar to the NACA0012 airfoil, the grids are refined near the leading edges and the trailing edges of each component of the three-element airfoil where the curvature varies greatly. Obviously, this mesh distribution is more suitable for CFD simulations.

### Treatment of special circumstances

The data sets used to train the model of ANN are relatively standard meshes generated by some traditional unstructured mesh generation methods. However, in the actual mesh generation process, the mesh quality may be inferior in some special cases, and the neural network had not been trained with these cases in the past, which may cause the neural network cannot correctly recognize these patterns. To solve this problem, it is necessary to perform coordinate translation processing on these mesh coordinate points to make it more similar to the characteristics of the data set for training the neural network. Experiments have verified that the modified method is effective.

**Figure 18:**
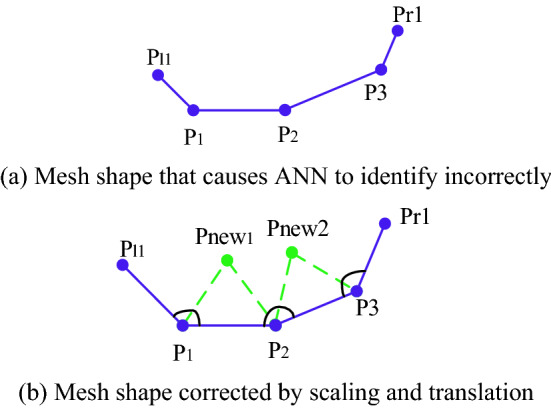
Special unreasonable geometric shape normalization processing.

As shown in Fig. 18a, this is just one case, and other cases are similar to it. The lengths of the four fronts are quite different. According to the method mentioned above, the first step is taking the length of Front [***P1***, ***P2***] as the reference value, and adjusting other fronts to make their lengths close to the reference length. In other words, the coordinates of ***P1*** and ***P2*** remain unchanged, and the positions of ***P***_***l1***_ and ***P3*** are changed by coordinate scaling. Finally, ***P***_***r1***_ is moved by coordinate translation to obtain its modified value. Through the series of operations, the shape in Fig. 18b is obtained, which is relatively standard input data.

### Mesh quality and time-consuming assessment

In this section, the quality assessment of isotropic meshes is discussed. The long-term CFD practice shows that mesh quality plays an important role in the accuracy of calculation results, so the mesh generated by any mesh generation method must be tested for quality. Here, we choose two criteria to quantify mesh quality. The first is index ***r*** which represents the ratio of a triangle area to its neighbors; the other one is index ***q***, which measures how similar a triangle is to an equilateral condition:

5 $$q_{i} = \frac{{4\sqrt 3 S_{i} }}{{a_{i}^{2} + b_{i}^{2} + c_{i}^{2} }}$$

where *S*_*i*_ is the area of triangle ***i***, and ***a***_***i***_, ***b***_***i***_, and ***c***_***i***_ are the three edge lengths of the triangle, respectively. The parameter ***q***_***i***_ varies between 1 (equilateral triangle) and 0 (degenerated triangle), the closer ***q***_***i***_ is to 1, the better mesh quality is.

As shown in Table 1, based on these two mesh quality testing criteria, the average and minimum values of these two indicators are calculated here. The present method is compared with commercial software ‘Pointwise’, experimental results show that the mesh quality generated by this method can reach the level of commercial software.

**Table 1:** Quality statistics of 2D triangular meshes.

Case	Software	min.***r***	ave. ***r***	min.***q***	ave. ***q***
Special characters	ANN-ADFT	0.358	0.862	0.624	0.965
	Pointwise	0.348	0.853	0.608	0.931
NACA0012	ANN- ADFT	0.431	0.852	0.736	0.927
	Pointwise	0.435	0.827	0.635	0.914
30P30N	ANN- ADFT	0.326	0.842	0.732	0.962
	Pointwise	0.317	0.826	0.645	0.968

On the other hand, the purpose of this method is to improve the efficiency of mesh generation, some unnecessary intersection checks are eliminated and two or more triangles are generated in each advancing step. As shown in Table 2, the computation time of the above three meshes generated by the traditional AFM, the single front ANN-based AFM, and the ANN-based ADFM are compared. The number of intersection checks of ANN-based ADFM is only half of the traditional AFM. In terms of mesh generation efficiency, the ANN-based ADFM is almost twice that of traditional AFM.

**Table 2:** Comparison of the time consumed by different methods to generate meshes.

Case	Number of intersection checks			Time cost/s		
	AFM	ANN-AFM	ANN-ADFM	AFM	ANN-AFM	ANN-ADFM
Special characters	201,196	134,805	101,104	13.98	9.78	7.02
NACA0012	156,703	109,419	85,346	10.89	5.25	4.15
30P30N	293,079	187,181	146,012	20.36	14.26	11.24

Finally, the statistics of the number of various patterns in the grid generation procedure is helpful to further analyze the internal reasons for the efficiency improvement. As shown in Table 3, the number of patterns 1 and 5 accounts for more than 50 percent of the total. Therefore, it is very important to deal with these patterns well to improve the efficiency of grid generation.

**Table 3:** The statistics of the number of various patterns in the grid generation procedure.

Case	Pattern 1	Pattern 2	Pattern 3	Pattern 4	Pattern 5	Pattern 6	Pattern 7	Pattern 8	Pattern 9	Pattern 10
Special characters	884	460	230	137	747	71	39	25	476	26
NACA0012	680	358	192	106	580	52	28	18	374	20
30P30N	1352	681	359	209	1104	45	51	34	704	37

## Conclusions

In this work, a novel automatic triangular mesh generation method is proposed for complex 2D geometries, which combines the most favorable advantages of ANN and AFM. The BP neural networks are established to identify external patterns during mesh generation. The method of automatic extraction and preprocessing of neural network training data set is introduced in detail. Finally, some typical 2D geometries are tested to validate the capability of the proposed method. The mesh generated by the new method has shown that the ANN can accurately classify external patterns to reduce the large number of intersection checks introduced by the traditional AFM. The mesh generation efficiency of the new method is twice that of the traditional AFM. We can imagine that the present double-front advancing process can be extended directly to multi-front advancing cases, then the mesh generation efficiency will be further improved. We will try it in our future work.

As with most supervised learning approaches, there is a trade-off between the time spent in the training phase of the machine learning algorithm and the reduction of large user interactions with traditional methods. Nonetheless, this can be a worthwhile expense when many solutions are required to deal with a large number of related problems, such as design and optimization issues. On the other hand, ANN outputs inevitably suffer from pattern misclassification in some extreme cases, which leads to the failure of grid generation. Therefore, after generating a new mesh, it is necessary to make an error judgment to verify whether the mesh generation is successful. If it fails, the grid is regenerated using traditional local reconstruction methods, and the neural network will be retrained to improve its adaptability.

After decades of CFD development in research and engineering techniques, a large amount of mesh data has been obtained with the empirical knowledge of researchers. How to design more advanced AI methods combined with mesh generation is our final goal. On the other hand, mesh generation technology in 2D is relatively mature, the next step is to extend this method to surface mesh generation and volume mesh generation in 3D, especially for viscous flow simulations.

## Data Availability

The datasets generated during and/or analyzed during the current study are available in the Github repository, https://github.com/lupeng396/ADFM.git.
